# Team-based infection preventionist review improves interrater reliability in identification of hospital-acquired infections

**DOI:** 10.1017/ash.2023.366

**Published:** 2023-09-29

**Authors:** Alyssa Castillo, Sarah Totten, Larissa Pisney

## Abstract

**Background:** The University of Colorado Health (UCHealth) metropolitan region is composed of 4 hospitals. Therein, 10 infection preventionists (IPs) retrospectively review all cases of potential central-line–associated bloodstream infection (CLABSI), catheter-associated urinary tract infection (CAUTI), and surgical site infection (SSI) to adjudicate whether each case meets the NHSN definitions for hospital-acquired infection (HAI). In August 2021, the UCHealth IP team structure transitioned from a subject-matter expert model (in which each IP reviewed a specific HAI) to a unit-based model (in which each IP reviewed all HAIs and SSIs on their assigned units) to create redundancy in knowledge and skill. The IP team subsequently instituted a weekly meeting to review all potential cases of HAI. We hypothesized that this review structure would result in increased consistency in the application of NHSN definitions across the UCHealth hospitals and units. **Methods:** From August 17, 2022, through March 3, 2023, the UCHealth IPs, managers, and medical directors met weekly for 1 hour via teleconferencing. Each IP presented key details for all near-miss and confirmed cases of SSI or HAI on their respective units and received questions and feedback from their peers and medical directors. Case determination was based on team discussion and consensus. If there was discordance in the interpretation of an NHSN case definition, a formal inquiry was sent to resolve the uncertainty. The number of cases reviewed, case determinations changed, and formal inquiries to NHSN were tracked. **Results:** During the study period, the IP team convened weekly meetings and reviewed 248 patient cases—of which 208 (83.9%) were confirmed HAIs. Based on collaborative team discussion, 14 cases (5.6%) were changed from reportable to nonreportable. Three cases (1.2%) originally thought to be nonreportable were changed to reportable. The HAI determination of a reportable case (eg, revision of a “superficial” SSI to “deep” SSI) was changed for 9 (6.0%). Following team discussion, 13 formal inquiries were sent to the NHSN to clarify case definitions, and these responses were collated for future reference. **Conclusions:** Team-based IP review of HAI cases improves consistency in application of NHSN case definitions and highlights areas of uncertainty in their interpretation. This team-based model of case review is a useful educational and practical tool to increase interrater reliability in case adjudication across large teams of IPs, to create a systematic way to query NHSN, and to ensure that knowledge gained is disseminated for future benefit.

**Disclosures:** None

